# Treatment with Depot Leuprolide Acetate in Girls with Idiopathic Precocious Puberty: What Parameter should be Used in Deciding on the Initial Dose?

**DOI:** 10.4274/jcrpe.galenos.2019.2019.0060

**Published:** 2020-03-19

**Authors:** Doğuş Vurallı, Ayfer Alikaşifoğlu, İrem İyigün, Dicle Canoruç, Alev Ozon, Nazlı Gönç, Nurgün Kandemir

**Affiliations:** 1 Hacettepe University Faculty of Medicine, Department of Pediatrics, Division of Pediatric Endocrinology, Ankara, Turkey; 2 Hacettepe University Faculty of Medicine, Department of Pediatrics, Ankara, Turkey

**Keywords:** Central precocious puberty, leuprolide, GnRH, GnRH analogue, gonadotropin releasing hormone agonist, precocious puberty, puberty

## Abstract

**Objective:**

Doses of gonadotropin releasing hormone (GnRH) analogues used to treat idiopathic central precocious puberty (iCPP) vary among clinicians. Study aims were to evaluate the efficacy of a monthly 3.75 mg dose of leuprolide acetate (LA) to suppress the hypothalamo-pituitary-gonadal (HPG) axis in girls with iCPP and to determine factors that may have an impact on the supressing dose.

**Methods:**

Study subjects were 220 girls receiving LA for iCPP. LA was started at a dose of 3.75 mg/28 days. Suppression was assessed using the GnRH test at the third month. To assess clinical suppression signs and symptoms of puberty were also evaluated. The dose of LA was increased to 7.5 mg/28 days in those who had a peak luteinising hormone (LH) ≥2 IU/L and in whom adequate clinical suppression of puberty was absent. Receiver operating characteristic curves were used to determine thresholds for clinical and hormonal factors affecting the suppressing dose of LA. Logistic regression analyses were used to investigate thresholds which might differentiate between those requiring high dose for suppression and those in whom lower dose LA was adequate.

**Results:**

Peak stimulated LH <2 IU/L was achieved in 88.6% with a dose of LA of 3.75 mg (0.11±0.03 mg/kg). Significant variables for differentiating the two doses were body weight (Wt) of 36.2 kg and/or body mass index (BMI)-standard deviation scores (SDS) of 1.64 (p<0.001). Multiple logistic regressions showed that Wt and BMI-SDS values above thresholds indicated requirement of LA at a dose of 7.5 mg/28 days (p<0.001).

**Conclusion:**

Monthly injections of 3.75 mg LA is an effective treatment in the majority of girls with iCPP. However, a higher initial dose may be preferred in patients with a Wt ≥36 kg or BMI-SDS ≥1.6 for effective suppression of the HPG axis.

**What is already known on this topic?**

Insufficient suppression due to inadequate dose of gonadotropin releasing hormone analogues (GnRHa) in central precocious puberty (CPP) may result in continued advancement of bone limiting final height whereas unnecessarily high doses may increase the risk of side effects, as well as total treatment costs. Monthly GnRHa injections are administered at different doses in different countries. For leuprolide acetate (LA), lower doses (3.75 mg/28 days, 80-120 μg/kg/28 days) are preferred in Europe and Asia, while higher doses (7.5-15 mg/28 days, 200-300 μ/kg/28 days) are used in the United States of America.

**What this study adds?**

LA treatment at doses of 3.75 mg/28 days is effective in suppressing the hypothalamo-pituitary-gonadal (HPG) axis in the majority of girls with idiopathic CPP. Higher initial doses may be preferred in patients with a body weight ≥36 kg or body mass index-standard deviation scores ≥1.6 for effective suppression of HPG axis.

## Introduction

The aim of gonadotropin releasing hormone (GnRH) analogue (GnRHa) treatment in central precocious puberty (CPP) is to allow normal growth, enabling a normal adult height and relieve psychosocial stress associated with early puberty (1,2,3). The intended long-term goals in such treatment include suppression of bone advancement and attainment of an age appropriate growth rate, in order to achieve a normal adult height parallel to target height (1,4). While short-acting nasal and daily injectable forms of GnRHa have been used previously, currently long-acting (monthly) or very-long-acting (three monthly) depot formulations or yearly implants that facilitate adherence to treatment are more commonly preferred (1,3,5). Insufficient suppression due to an inadequate dose of GnRHa may result in continued advancement of bone age (BA) thus limiting final height whereas unnecessarily high doses may increase the risk of side effects, as well as total treatment costs. Higher doses have been shown to suppress both growth and bone mineral accrual rates (6,7). The doses of GnRH analogues used in CPP may vary with clinican preference, as well as local regulatory approvals. Monthly GnRHa injections are administered in different doses in different countries. For leuprolide acetate (LA), lower doses (3.75 mg/28 days, 80-120 µ/kg/28 days) are preferred in Europe and Asia (8,9,10), while higher doses (7.5-15 mg/28 days, 200-300 µ/kg/28 days) are used in the United States of America (11). In the face of such dosage variation, the best dose for optimal pituitary desensitization during monthly leuprolide treatment is still a matter of discussion. The aim of this study was to evaluate the efficacy of a monthly 3.75 mg dose of LA to suppress the hypothalamo-pituitary-gonadal (HPG) axis in girls with idiopathic CPP (iCPP) and to determine factors that may have an impact on the supressing dose. We also aimed to define the best predictor among these factors for the optimal initial dose of LA.

## Methods

A total of 220 girls with a diagnosis of iCPP who were followed between January 2012 and January 2018 who had received 3.75 mg LA (Lucrin depot, subcutaneous or intramuscular) once every 28 days were evaluated. Age at diagnosis, BA, body weight (Wt), height, pubertal stage, basal estradiol levels, basal and stimulated gonadotropin levels, pelvic ultrasonography and magnetic resonance imaging (MRI) findings of the pituitary gland were recorded. CPP was diagnosed based on breast development being at Tanner stage 2 or higher before eight years of age, and peak luteinizing hormone values ≥5 IU/L during the GnRH test (12). A GnRH test was performed in all patients at the time of diagnosis. Blood samples were collected at baseline (zero minutes) for follicle stimulating hormone (FSH) and LH measurements. Then the patients were intravenously administered 100 µg/m^2^ of GnRH (gonadorelin acetate, Ferring^®^). Following drug administration, blood samples were collected at 20, 40, 60, and 120 minutes for FSH and LH measurement (13). With the exception of four patients presenting with menarche, all patients were followed for 3-6 months before the treatment decision. GnRHa treatment was given to patients with progressive CPP, determined according to the following criteria: a) Growth velocity above 6 cm/year; b) Advanced BA defined as BA ≥2 years compared with chronological age; c) Rapid progression in pubertal stages defined as progression of puberty from one stage to the next in less than six months; and d) Decrease in predicted adult height compared to target height (14). Pituitary MRI was performed in all cases and the underlying organic pathology was investigated. Cases with no pathological MRI findings were considered to be idiopathic and were included in the study. Subjects were excluded from the analysis if they had any additional conditions that might affect puberty onset such as hypothyroidism, growth hormone deficiency or congenital adrenal hyperplasia. LA was started at an initial dose of 3.75 mg/28 days for all patients with iCPP. For all patients who were started on GnRHa treatment, the GnRH test was repeated in the third month of treatment and the HPG axis was considered to be suppressed if peak LH levels were <2 IU/L (15,16,17). Clinical signs and symptoms of puberty were also evaluated every 3-6 months to determine whether pubertal suppression was achieved clinically. Parameters of good clinical control included stabilization or regression of pubertal findings, decrease in height velocity to prepubertal levels, cessation of BA progression, and improvement in final height prediction. The dose of LA was increased to 7.5 mg/28 days in those who have a peak LH ≥2 IU/L and in whom clinical suppression of puberty was not achieved. All patients who had a peak LH ≥2 IU/L in the third month GnRH test did not have adequate clinical suppression of puberty and dose LA dose was increased in all of these cases. The higher dose was similarly tested with GnRH test for appropriate suppression of HPG three months later. We compared clinical and hormonal characteristics of the two populations whose HPG axis was suppressed either with 3.75 mg/28 days or 7.5 mg/28 days of LA. Follow up included clinical and hormonal evaluation of all patients every six months after the initial treatment and, during long-term follow-up continuous clinical and hormonal suppression was observed.

### Auxological Parameters

Body Wt were measured with a digital body weighing scale and heights were measured in the standing position with a Harpenden stadiometer by a nurse trained in height measurements and auxology. The percentile curves of the Centers for Disease Control and Prevention (CDC) were used to interpret the growth data (18). Height standard deviation scores (SDS) for chronological age and BA were calculated using CDC charts. Body mass index (BMI) was calculated using the standard equation (Wt in kg/height in meters squared). BMI-SDS was calculated according to the LMS method using CDC charts (19). Puberty staging was evaluated using Marshall and Tanner staging (20). The BA was evaluated according to the Greulich and Pyle atlas (21).

### Hormone Assays

The immunochemiluminometric assay method using commercial kits (ARCHITECT System, Abbott Laboratory Diagnostics, USA) were used to measure FSH, LH and estradiol levels. The sensitivity of the FSH, LH, and estradiol assays was 0.3 IU/L, 0.07 IU/L, and 10 pg/mL respectively.

### Ethics Statements

The study protocol was approved by the Ethics Commitee of Hacettepe University (approval number: GO 19/453-41). The requirement for informed consent was waived due to the retrospective nature of the study.

### Statistical Analyses

Statistical analyses were performed using the Statistical Package for Social Sciences software package for Windows (version 19.0; SPSS Inc., Chicago, IL, USA). Testing for normality was performed by Shapiro-Wilk test and the data was found to be normally distributed. Data are shown as mean ± standard deviation values. Student’s t-test was used in comparisons of independent samples. Receiver operating characteristic (ROC) curves were used to determine threshold levels for factors with an impact on the dose of LA that suppressed HPG axis (age, body Wt, BMI, BMI-SDS, basal LH, basal estradiol, peak stimulated LH). Threshold values were analyzed to investigate if they differentiated the two populations of patients whose HPG axis was suppressed either with 3.75 mg/28 days or 7.5 mg/28 days of LA using univariate logistic regression. Pubertal stages were grouped into early (Tanner 2 and 3) vs advanced (Tanner 4 and 5), and impact of pubertal stages on suppressing doses of LA were also analyzed. Statistically significant factors in univariate analysis were re-evaluated using multiple logistic regression analysis. A p value of less than 0.05 was considered statistically significant.

## Results

Peak stimulated LH was <2 IU/L after three months of treatment in 88.6% (195/220) of the patients with the initial LA dose of 3.75 mg/28 days. In the remaining 11.4% (25/220), the LA dose was increased to 7.5 mg/28 days, as puberty suppression was not achieved clinically and hormonally. The GnRH test was repeated in patients who received 7.5 mg/28 days at the third month of dose escalation. The peak LH levels were found to be <2 IU/L in all patients and hormonal puberty suppression was achieved in all of them. Regression in the clinical signs and symptoms of puberty and cessation in BA progression were observed. Growth rates decreased to prepubertal levels in all patients with successful hormonal suppression. Consequently, suppression of the HPG axis was achieved in all patients by the sixth month of treatment (Table 1).

Among cases that achieved HPG suppression at the dose of 3.75 mg LA/28 days, the pubertal stage at the time of diagnosis was Tanner stage 2 in 35.9% (70/195), Tanner stage 3 in 54.4% of cases (106/195), and Tanner stage 4 in 9.7% (19/195). Among the cases with successful suppression with a dose of 7.5 mg LA/28 days, 60% (15/25) were at Tanner stage 3, 24% (6/25) were at Tanner stage 4 and 16% (4/25) were at Tanner stage 5 at the time of diagnosis. These latter four patients presented with menarche. There were no cases presenting with menarche among the patients whose puberty were suppressed with 3.75 mg LA. The stage of puberty at the time of diagnosis was significantly advanced among patients for whom the effective dose was 7.5 mg (p<0.001). Suppression was achieved with LA 3.75 mg/28 days in all patients (70/70) who were at Tanner stage 2, in 87.6% of patients (106/121) at Tanner stage 3 and 76% of patients (19/25) at Tanner stage 4 at the time of diagnosis, while all patients (4/4) at Tanner stage 5 required 7.5 mg LA for the suppression of the HPG axis.

A comparison of the clinical and laboratory findings at the time of diagnosis of the patients for whom HPG axis suppression was achieved with 3.75 mg and 7.5 mg LA dosages revealed that those requiring 7.5 mg LA for suppression were found to have higher mean body Wt, BMI and BMI-SDS values and also elevated mean baseline LH, estradiol and peak stimulated LH levels at the time of diagnosis (Table 1). Among the patients with successful suppression at a dose of 3.75 mg LA, suppression was achieved with a mean dose of 0.11±0.03 mg/kg, whereas in the patients for whom 3.75 mg dose was not adequate for suppression, the initially given dose of 0.08±0.02 mg/kg (3.75 mg in total) was insufficient due to high body Wt, and suppression was only achieved when these patients received a dose of 7.5 mg LA (0.16±0.03 mg/kg).

ROC curves were used to determine the threshold levels for the factors which may affect the dose that achieved pubertal suppression. The best threshold values that differentiated the two doses (3.75 mg/28 days vs 7.5 mg/28 days LA) were 36.2 kg for body Wt (AUC=0.934, p=0.0001, sensitivity 100%, specificity 66.7%), 20.7 kg/m^2^ for BMI [area under the curve (AUC)=0.964, p=0.0001, sensitiviy 94%, specificity 74%], +1.64 for BMI-SDS (AUC=0.914, p=0.0001, sensitivity 100%, specificity 71.2%), 1.5 IU/L for basal LH (AUC=0.710, p=0.0004, sensitivity 68%, specificity 67%), 41 pg/mL for basal estradiol (AUC=0.898, p=0.0001, sensitivity 100%, specificity 68%) and 17.6 IU/L for peak stimulated LH (AUC=0.710, p=0.0006, sensitivity 68%, specificity 67%) in ROC analysis. Age did not differ between the two different dose populations (8.2±1.0 vs 8.3±0.5). Univariate analysis indicated Wt, BMI and BMI-SDS above the defined thresholds, as well as advanced stage of puberty were associated with higher dose of LA for effective treatment (p<0.001, <0.001, <0.001, 0.02, respectively) (Table 2). However, thresholds for basal LH, estradiol and stimulated LH peak did not differentiate between the two doses of LA since they were insignificant in the univariate analysis. Since Wt and BMI-SDS were related factors, these factors were not used together in multiple regression analysis but tested in separate regression models. Multiple logistic regression showed that thresholds for BMI-SDS and Wt were significant to differentiate the two doses of LA (p<0.001) (Table 3, 4), whereas thresholds for basal LH, estradiol and stimulated peak LH did not differentiate the two dose groups and thus could not be used to assess dose of LA required to suppress puberty.

## Discussion

In this study we showed that LA treatment at a dose of 3.75 mg/28 days was effective in suppresing the HPG axis in the majority (88.6%) of girls with iCPP, while suppression was achieved in the remaining 11.4% of cases with a dose of 7.5 mg/28 days. Studies from Europe and Brazil have shown that suppression of the HPG axis can be achieved in 85-96% of the cases using a dose of 3.75 mg/28 days LA (7,9,22,23) which is consistent with our findings. Studies carried out in the United States report higher LA doses of at least 7.5 mg/monthly for HPG suppression (24,25). In Japan, Tanaka et al (26) compared doses of 10, 30 and 90 µg/kg in 36 children with CPP (90 µg/kg being roughly equal to 3.75 mg LA) and concluded that the minimum suppressive dose of LA was 30 µg/kg, which is one tenth of the US recommendations and much lower than the dose of 3.75 mg/28 days.

Recently, use of three-monthly LA depot preparations in pediatric patients appeared in the literature (27). The dose difference in the use of LA depot formulations between United States and Europe was reported to persist in this report. In a French study of 40 cases with CPP, a three-monthly dose of 11.25 mg provided suppression of GnRH-stimulated gonadotropin levels (28). In a study from the United States, Fuld et al (29) compared three doses of LA (LA 7.5 mg/month, 11.25 mg/3 months and 22.5 mg/3 months) in 54 patients with CPP, and showed that the dose of 22.5 mg/3 months provided a better suppression of LH levels in comparison to a dose of 11.25 mg/3 months. However, these last two doses did not differ in their effect on other parameters studied which were growth velocity, progression of BA or estradiol levels. Mericq et al (30) compared the same three doses of LA in 14 children and recommended the use of high-dose LA depot formulations in cases with a body Wt of more than 30 kg, although LA depot formulation at a dose of 11.25 mg/3 months also provided sufficient (75%) pubertal suppression.

One major constraint in the published studies is that they were carried out in small populations of children. What is more, many studies analyzed mixed populations with respect to sex, involving both girls and boys, and etiology which included both idiopathic and organic cases. The GnRHa dose required to suppress the HPG axis may differ between girls and boys, and also between CPP cases of organic or idiopathic etiology. In addition most studies comparing monthly vs three monthly preparations did not include LA at a dose of 3.75 mg/28 days.

There is one study from the USA which included monthly 3.75 mg LA and compared it with 7.5 mg/month and 11.25 mg/3 months LA. In that study Badaru et al (27) showed that in patients on treatment with LA using a dose of 3.75 mg/month and 11.25 mg/3 months had peak stimulated LH and FSH levels higher than those using a dose of 7.5 mg/month (the mean depot LA-stimulated LH was 1.30±0.74, 1.73±0.99 and 2.13±1.41, with doses of 7.5 mg/month, 3.75 mg/month, and 11.25 mg/3 months, respectively). However, the authors underlined that clinically significant elevation to merit dose escalation was observed only in a small number of patients. In addition, serum estrogen levels did not differ between the three dose regimens.

In the current study a large homogenous population of girls with iCPP was analyzed to see if pubertal suppression can be achieved with lower monthly doses of LA. The suppressive dose of LA and factors that may impact on its effectiveness were also investigated. It was hypothesized that such an analysis may help predict the dose of LA that can suppress puberty and avoid high doses of LA thus avoiding the associated adverse effects. The current study suggests that LA at a dose of 3.75 mg/28 days effectively suppresses HPG axis in most girls with iCPP. A comparison of the two populations (pubertal suppression by LA 3.75 mg/28 days vs 7.5 mg/28 days) showed that there was significant difference between them in several clinical and laboratory parameters such as body Wt, BMI, basal LH, estradiol, and peak stimulated LH on the initial GnRH test. As would be expected, higher GnRHa doses may be required for pubertal suppression in cases at advanced stages of puberty. Similarly, the patients who required dose escalation had higher baseline LH and estradiol levels as well as higher peak LH in the GnRH test. We showed that the most significant factors indicating a need for LA at a dose of 7.5 mg/28 days were body Wt ≥36.2 kg, and BMI-SDS ≥1.64.

In general, the use of high dose of GnRHa may have two important consequences. Firstly, oversuppression of puberty using a high dose of GnRHa may carry the risk of suppression of growth. Secondly, extensive pubertal suppression may affect bone mineral density (BMD) adversely, since long-term oversuppression of estrogen may decrease bone mineral accrual. In addition, higher doses would increase treatment costs excessively. It is well-known that one major purpose of GnRHa treatment is to increase the final height potential. Thus it may seem contradictory to suggest that oversuppression of puberty may adversely affect growth. Studies investigating long-term effects of GnRHa treatment have shown the expected deceleration in BA advancement as well as suppression of puberty increasing final height (31). However, these studies did not address the relation between height gain and the dose of GnRHa used to suppress puberty.

Mitamura et al (32) studied 24 hour gonadotropin and sex steroid profile in 17 girls (5-11.5 years) and showed that a diurnal rhythm of gonadotropins was present in all subjects including those aged 5-6 years. Also one third of their prepubertal subjects had elevated early morning stradiol. They suggested that preparation for the onset of female puberty may begin in 5- to 6-year-old girls. Lampit et al (33) compared GnRHa therapy with and without mini dose estrogen in a small number of patients. They showed that during GnRH agonist therapy a mini-dose of estrogen effectively maintained normal prepubertal growth without acceleration of bone maturation for at least 24 months, whereas growth velocity may decrease in those receiving GnRHa alone.

Currently it is not clear whether over suppression of the HPG axis would do more harm than good in terms of growth, since this issue is not specifically addressed. Moreover it is not known whether such an oversuppression, even if it decreased growth velocity, would also affect final height adversely. Unfortunately, long-term results of high dose LA (7.5 mg/28 ds or higher) are scarce, and no study has compared long term height gain with low versus high dose LA. Extensive suppression of growth may be unwanted. Thus studies are required to specifically address these issues.

Puberty is the critical period for bone development and accrual of peak bone mass (34). Approximately half of peak bone mass is acquired during puberty (35). Postmenopausal decrease in BMD, as well as reduction of BMD in premenopausal adults using GnRHa treatment is attributed to hypo-estrogenism. GnRHa therapy for CPP is also suggested to create a hypo-estrogenic condition which may have a negative impact on bone mass (35). There are contradictory reports to that effect in children. Some studies report a decrease in BMD in children using GnRHa therapy, whereas in others no difference was shown during treatment (36,37). Most studies were carried out using 3.75 mg/28 days of LA. Oversuppression of HPG axis with 7.5 mg/28 days or higher doses may have a greater negative impact on accrual of bone mineral. There is a need for long-term, randomized trials investigating the impact of high dose of LA on bone health in children with CPP.

Another disadvantage of unnecessary high-dose LA treatment is that it is costly. Healthcare costs have been increasing globally in the last decades, and there is an increasing pressure worldwide to reduce costs and improve efficiency, while maintaining quality. Thus expensive treatments without added benefit to health is an issue of consideration.

The present study had several advantages in terms of its sample size and choice of patient population. It also provided an analysis of factors that may affect the suppressive dose of LA in a large sample of 220 girls with iCPP, and provides a strategy based on body Wt in the choice of the initial dose of LA for pubertal suppression. Another advantage was the use of the gold standard GnRH test to assess pubertal suppression.

### Study Limitations

Dose titration was not carried out in this study. LA was used at a dose of 3.75 mg initially and 7.5 mg subsequently in those with inadequate suppression. This approach does not provide information on minimum effective dose for successful suppression.

## Conclusion

Monthly injections of LA (3.75 mg/28 days) was an effective treatment in terms of HPG axis suppression in the majority of girls with iCPP. This treatment option was also more cost-effective than an initial high dose of 7.5 mg/28 days dose. A higher initial dose may be preferred in patients with a body Wt ≥36 kg or BMI-SDS ≥1.6 for effective suppression of the HPG axis although these patients would require closer clinical follow-up. Further studies comparing long term impact of different doses of GnRHa on growth and bone health are required.

## Figures and Tables

**Table 1:**
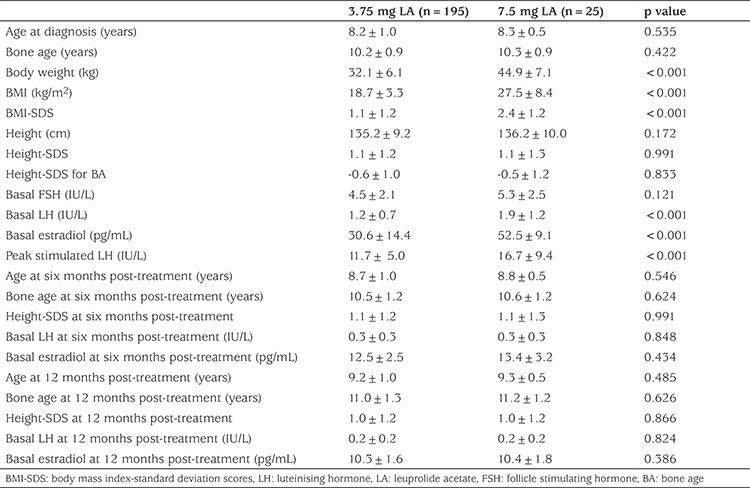
Clinical and laboratory characteristics of patients treated with leuprolide acetate at doses of 3.75 mg vs 7.5 mg

**Table 2:**
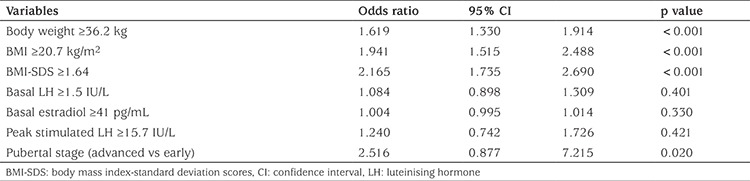
Factors affecting treatment dosage based on univariate logistic regression analysis

**Table 3:**
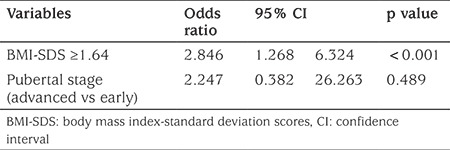
Factors affecting treatment dose based on multivariate logistic regression analysis (first model)

**Table 4:**
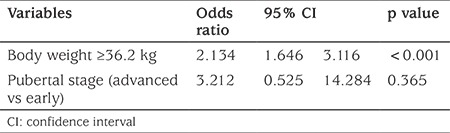
Factors affecting treatment dose based on multivariate logistic regression analysis (second model)
